# Supplementary material for: Exchange rate sensitivity influencing the economy: The case of Sri Lanka
Source: PLoS One. 2022 Jun 16;17(6):e0269538. doi: 10.1371/journal.pone.0269538 (PMC9202912; doi:10.1371/journal.pone.0269538)
Supplement: Attachment — Submitted filename: Response to Reviewers.docx [file pone.0269538.s001.docx]

**Point–by–point response to reviewers**

Dear editor and reviewer.

Greetings. Thank you very much for the fruitful comments.

Please note that page numbers and line numbers refereed in this document is align with the revised manuscript which has track changes.

| **Comments of Reviewers** | **Comments of Authors** |
| --- | --- |
| **Comment 1** |  |
| **Is the manuscript technically sound, and do the data support the conclusions?**  The manuscript must describe a technically sound piece of scientific research with data that supports the conclusions. Experiments must have been conducted rigorously, with appropriate controls, replication, and sample sizes. The conclusions must be drawn appropriately based on the data presented.  Reviewer 01 – Partly | Noted with thank you. This has been incorporated in the revised manuscript with track changes. |
| **Comment 2** |  |
| **Has the statistical analysis been performed appropriately and rigorously?**  Reviewer 01 – Yes | Well noted. Thank you for your comment. |
| **Comment 3** |  |
| **Have the authors made all data underlying the findings in their manuscript fully available?**  The PLOS Data policy requires authors to make all data underlying the findings described in their manuscript fully available without restriction, with rare exception. The data should be provided as part of the manuscript or its supporting information or deposited to a public repository. For example, in addition to summary statistics, the data points behind means, medians and variance measures should be available. If there are restrictions on publicly sharing data—e.g., participant privacy or use of data from a third party—those must be specified.  Reviewer 01 – Yes | Noted with thank you. |
| **Comment 4** |  |
| **Is the manuscript presented in an intelligible fashion and written in standard English?**  PLOS ONE does not copyedit accepted manuscripts, so the language in submitted articles must be clear, correct, and unambiguous. Any typographical or grammatical errors should be corrected at revision, so please note any specific errors here.  Reviewer 01 – No | Thank you and well noted. This has been in cooperated in the revised manuscript with track changes. |
| **5. Review Comments to the Author**  **Reviewer** | |
| **Comment #1**   There are many grammatical errors and/or typos and/or repetitions and/or missing plurals: "This present reseach", "not used earlied used in Sri Lankan studies", "the ability borrow", "Foreign direct investment", "one of the most discussed microeconomic variables", "foreign reserve", "explored the determinates", "could cause deter" etc. I think that the whole paper should linguistically revised; | Thank you and well noted. This has been in cooperated in the revised manuscript with track changes.   - "This present reseach" - rectified in Line #23. - "not used earlied used in Sri Lankan studies" rectified line # 41. - "the ability borrow” - rectified line #73. - "Foreign direct investment" rectified line #71. - "one of the most discussed microeconomic variables" – removed – line # 192. - "foreign reserve" - rectified in line # 252, 253, 268, 269. - "could cause deter" – rectified Line #619.   The specifically highlighted errors have been corrected. In addition, revised version has been proofread. Correction has been made including typos, comma correction and brevity. |
| **Comment #2**   The contrast between some parts of the paper and others is particularly strong. On the one hand, the authors define macroeconomic concepts, which are rather known and unnecessary ("Exchange rate or conversion rate simply means the..." or "The exchange rate volatility means that the prices of imports and exports fluctuate" or "The reason for selecting USD to analyse the exchange rate is because the majority of international trade is conducted as USD"). On the other, they all of the sudden run several econometrical tests which are in turn particularly complex. The jump from one approach to another is - in my opinion - too pronounced and several, rather self-evident definitions should be cut and/or better articulated; | Thank you and well noted. This has been in cooperated in the revised manuscript with track changes.   - "Exchange rate or conversion rate simply means the..." – removed Line #47-48. - "The exchange rate volatility means that the prices of imports and exports fluctuate" – removed. Line # 78 -82. - "The reason for selecting USD to analyse the exchange rate is because the majority of international trade is conducted as USD – Removed. Line # 98-101. |
| **Comment #3**  I don't really understand why the authors have to create a complex research scheme based on several papers (whose "suitability" as explained in Fig. 1 cannot be externally verified), although the macroeconomic factors identified as traditionally explaining exchange rate volatility are commonly accepted and do not need to be really justified. For instance, the relationship between interest rates or inflation and exchange rates has been widely analysed in the economic literature, meaning that it is not necessary to base the authors' decision on some papers retrieved in electronic journal databases. Moreover, I am pretty sure that much more articles have been written on these topics than those selected. Therefore, the parts from page 5 to 10 are - at least, in my opinion - not necessary or should be reduced so that the basic ideas (i.e., why the authors will analyse specific macroeconomic factors) are conveyed without getting too much into the details of the papers (which mention other countries than Sri Lanka (i.e., are therefore distracting)); | Thank you for your informative feedback. We agree with your statement. Literature review has been revised in the manuscript with track changes from Line #113 to 305 only to include Sri Lankan related studies and not to delve in too much detail of each study. |
| **Comment #4**  I feel that the authors should add graphs (for instance, with data from World Bank databases) showing the evolution of the macroeconomic factors analysed for Sri Lanka during the time period of analysis. For instance, the exchange rate LKR/USD, the inflation rate etc.; | Thank you and well noted. We would like to incorporate World Bank data however World Bank database only provides Inflation either on an Annual basis ([URL1](https://data.worldbank.org/indicator/FP.CPI.TOTL.ZG?locations=LK)) or quarterly basis ([URL2](https://databank.worldbank.org/reports.aspx?source=global-economic-monitor-%28gem%29)). Since we analysed variables on a monthly basis, we were unable to incorporate World Bank data. Therefore, we were limited to only Monthly data from Central Bank of Sri Lanka and have accordingly created graphs of all the analysed variables in Page No 17. |
| **Comment #5**  the authors should better explain the added value of their research. What is new? What is particular? These are mentioned in the "Objective", but should be better highlighted also in the "Conclusion"; | Thank you and well noted. This has been in cooperated in the revised manuscript with track changes from Line # 653 to 663. |
| **Comment #6**  some assertions should be better clarified by means of economic literature. For instance, from "Although numerous studies..." to "fluctuation in another country". | Thank you and well noted. With regards to “numerous countries” relevant example and explanation is given from Line # 50 to 58  With regards to "fluctuation in another country" relevant example and explanation is given from Line # 63 - 67 |
